# Activation of Blood CD3^+^CD56^+^CD8^+^ T Cells during Pregnancy and Multiple Sclerosis

**DOI:** 10.3389/fimmu.2017.00196

**Published:** 2017-02-23

**Authors:** Clara de Andrés, Lidia Fernández-Paredes, Marta Tejera-Alhambra, Bárbara Alonso, Rocío Ramos-Medina, Silvia Sánchez-Ramón

**Affiliations:** ^1^Department of Neurology, Hospital General Universitario Gregorio Marañón, Madrid, Spain; ^2^Department of Clinical Immunology, IdISSC, Hospital Clínico San Carlos, Madrid, Spain; ^3^Department of Immunology, Hospital General Universitario Gregorio Marañón, Madrid, Spain; ^4^Department of Microbiology I, Complutense University School of Medicine, Madrid, Spain

**Keywords:** pregnancy, regulatory immune response, postpartum, NK cell, CD3^+^CD56^+^CD8^+^ cells, multiple sclerosis

## Abstract

A striking common feature of most autoimmune diseases is their female predominance, with at least twice as common among women than men in relapsing–remitting multiple sclerosis (MS), the prevailing MS clinical form with onset at childbearing age. This fact, together with the protective effect on disease activity during pregnancy, when there are many biological changes including high levels of estrogens and progesterone, puts sex hormones under the spotlight. The role of natural killer (NK) and NKT cells in MS disease beginning and course is still to be elucidated. The uterine NK (uNK) cells are the most predominant immune population in early pregnancy, and the number and function of uNK cells infiltrating the endometrium are sex-hormones’ dependent. However, there is controversy on the role of estrogen or progesterone on circulating NK (CD56^dim^ and CD56^bright^) and NKT cells’ subsets. Here, we show a significantly increased activation of CD3^+^CD56^+^CD8^+^ cells in pregnant MS women (MSP) compared with non-pregnant MS women (NPMS) (*p* < 0.001) and even with respect to healthy pregnant women (HP, *p* < 0.001), remaining increased even after delivery. The dynamics of expression of early activation marker CD69 on CD3^+^CD56^+^CD8^+^ cells showed a progressive statistically significant increase along the gestation trimesters (T) and at postpartum (PP) with respect to NPMS (1T: *p* = 0.018; 2T: *p* = 0.004; 3T: *p* < 0.001; PP: *p* = 0.001). In addition, early activation expression of CD69 on CD3^+^CD56^+^CD8^+^ cells was higher in MSP than HP in the first two trimesters of gestation (*p* = 0.004 and *p* = 0.015, respectively). NPMS showed significantly increased cytotoxic/regulatory NK ratio compared with healthy controls (*p* < 0.001). On the other hand, gender studies showed no differences between MS women and men in NK and CD3^+^CD56^+^CD8^+^ cells’ subsets. Our findings may add on the understanding of the regulatory axis in MS during pregnancy. Further studies on specific CD8^+^ NKT cells function and their role in pregnancy beneficial effects on MS are warranted to move forward more effective MS treatments.

## Introduction

Multiple sclerosis (MS) is a prototypic autoimmune inflammatory disorder of the central nervous system (CNS), in which both adaptive and innate immune systems are assumed to participate in demyelination and neurodegeneration (1). Epidemiological data indicate that relapsing–remitting-MS is more prevalent in females than in males (3–2:1) (2), while men tend to develop a more severe and chronic form of the disease. Several factors have been proposed to contribute to gender bias in susceptibility to MS. The fact that MS is typically diagnosed in the fertile age turns to sex hormones, an important focus of study. For female patients with MS, pregnancy is one of the strongest known modulators of disease activity (3, 4), largely attributed to elevated levels of circulating sex hormones, such as estrogen or progesterone.

Natural killer (NK) cells are innate immune cells with a major role in eliminating virus-infected and tumor cells. Current evidences on the role of peripheral NK cells in exacerbating or dampening autoimmunity responses in MS are controversial (5). Peripheral NK cells’ activity has been reported to be reduced in the setting of MS (6–8), although its role in the pathophysiology of the disease is largely unknown. On the other hand, the uterine natural killer (uNK) cells are the most predominant immune population at the decidua in early pregnancy and disclose a regulatory phenotype, while its origin remains to be determined. However, effects of sex hormones on the immune system, and more specifically on peripheral NK and NKT cells, have not been clearly elucidated.

Two major functionally distinct subsets of NK cells have been described based on the relative expression of the markers CD16 and CD56 (9). Typically, the majority (approximately 90%) of circulating NK cells have low expression of CD56 and high levels of CD16 and perforin, and display potent cytolytic activity (cytCD56^dim^). This subset can not only spontaneously lyse targeted tumor cells but also induce rapid inflammatory responses by releasing significant amounts of chemokines and proinflammatory cytokines when their activating receptors are engaged. By contrast, NK cells expressing high levels of CD56 and low CD16 are more abundant in secondary lymphoid tissues and tonsils (10, 11), and comprise nearly 70% of human decidual lymphocytes (9). Following monokine stimulation, CD56^bright^CD16^−^ proliferate and produce immunoregulatory cytokines, including IFN-γ, TNF-α, and GM-CSF, and there is a general consensus in the literature that ascribe them a protective role in the neuron-immunological context in MS (regCD56^bright^) (12).

NKT cells comprise a small subset of lymphocytes that possesses characteristics of both NK cells and conventional T cells, being able to release prototypical Th1 and Th2 cytokines after TCR ligation (13), and to induce perforin-, Fas-, and TNF-related cytotoxicity (14). Thus, these cells can have either protective or deleterious effects by promoting either inflammation or immune tolerance. Several studies have highlighted their regulatory role in autoimmune diseases, such as MS, type I diabetes mellitus, primary biliary cirrhosis, systemic lupus erythematosus, rheumatoid arthritis, psoriasis, and atherosclerosis, among others (15). NKT cells recognize lipid or glycolipid antigens presented by the MHC class I-related protein CD1d and exert their multiple functions, including antibacterial and antiviral immune responses, tumor-related immunosurveillance or immunosuppression, and inhibition or promotion of the development of autoimmune diseases (16). Few studies have revealed alterations in the numbers (17, 18) and functions of NKT cells in MS patients, despite MS is an organ-specific disease in which myelin lipids are a major target [reviewed in Ref. (19)]. The fact that specific NKT cells prevalence and function is restored in MS patients in remission after IFN-β treatment (12); that oral corticosteroids induce a Th2 bias in the cytokine profile of these cells (20); and that 1,25(OH)D3 vitamin induce protection from EAE in mice dependent of NKT cell-derived IL-4 (21) suggests that NKT cells might exert immunoregulatory more than detrimental effects in MS. Depletion of iNKT in mice show a more severe EAE course (22). On the contrary, expanding iNKT protects from EAE by suppressing Th1 and Th17 responses (23, 24). Type II NKT cells exert also protective effects on EAE models (25). Further, activation of NKT cells with synthetic lipid antigens protects mice against the development of MS-like disease [reviewed in Ref. (26)]. Very little is known about the CD8^+^NKT role in MS. Interactions with other immunoregulatory cell types, such as regulatory T cells and immunosuppressive myeloid cells, might exert immunoregulatory effects in MS by producing Th2 cytokines [reviewed in Ref. (20)]. Further, CD8^+^NKT cells can function as antigen-specific suppressive cells to regulate the immune response through killing antigen-bearing DCs (27) and efficiently suppress the proliferation and expansion of activated T cells (28).

In this study, we sought to determine, first, the distribution of NK and NKT-like cells subsets according to sex and their involvement in the disease and pregnancy. Second and given the role of uNK cells in maternal tolerance during pregnancy, we explored the distribution of the NK and NKT-like cell subsets during normal and MS pregnancy, to ascertain whether they exert a role during pregnancy. To address this goal, we evaluated the proportion and activation status (measured by CD69 expression) of circulating NK subsets (regCD56^bright^ and cytCD56^dim^) and CD3^+^CD56^+^CD8^+^ cells in both men and women from healthy subjects, MS patients, and pregnant women. Finally, we measured estrogens and progesterone levels in the luteal and follicular phases of the menstrual cycle of healthy and MS women to assess the effects of the menstrual cycle and gender on NK activation.

## Patients and Methods

### Subjects

A total of 124 subjects were studied. Among them, 70 MS patients, 30 non-pregnant women (mean age 39.0 years, range 28–51), 10 men (mean age 36.5 years, range 30–43), and 30 pregnant women (mean age 34 years, range 31–36) were consecutively recruited at the Unit of Multiple Sclerosis of the University General Hospital Gregorio Marañón of the Community of Madrid, Spain. All patients fulfilled definite MS diagnosis according to McDonald’s criteria (1). These patients had not received any immunomodulatory or immunosuppressive therapy in the previous 3 months and had non-active disease at the time of sample collection.

A group of 54 age-matched healthy controls were recruited from volunteers at University General Hospital Gregorio Marañón during the same period: 32 women (mean age 28.1 years, range 21–39), 9 men (mean age 31.6 years, range 21–40), and 13 pregnant women (mean age 33 years, range 30–37). All 32 healthy women included in the study had regular menstrual cycles and were studied at days 1–3 and at day 14 (ovulation) of their menstrual cycle. None of HC and MS female had received treatment with glucocorticoids and contraceptive pills prior to the study inclusion. Clinical and demographic characteristics of individuals enrolled in the study are summarized in Table 1. The Ethics Committee of the institution approved the protocol, and all subjects provided their written informed consent.

**Table 1:** **Clinical and demographical characteristics of multiple sclerosis (MS) patients and healthy controls (HC) included in the study: pregnant MS women (MSP), non-pregnant MS women (NPMS), MS men, healthy pregnant women (HP), non-pregnant healthy control women (NPHC), and HC men**.

	MSP	NPMS	MS men	HP	NPHC	HC men
No. of patients	30	30	10	13	32	9
Age (years)	34 (31–36)	39 (28–51)	36 (30–43)	33 (30–37)	28 (21–39)	31 (21–40)
EDSS	0 (1) (prior pregnancy)	1 (0–1.6)	1 (0.5–1.5)	NA	NA	NA

*Data are expressed as median (interquartile range)*.

*EDSS, Expanded Disability Status Scale*.

### NK and NKT Cell Subsets’ Analysis

**Figure 1:**
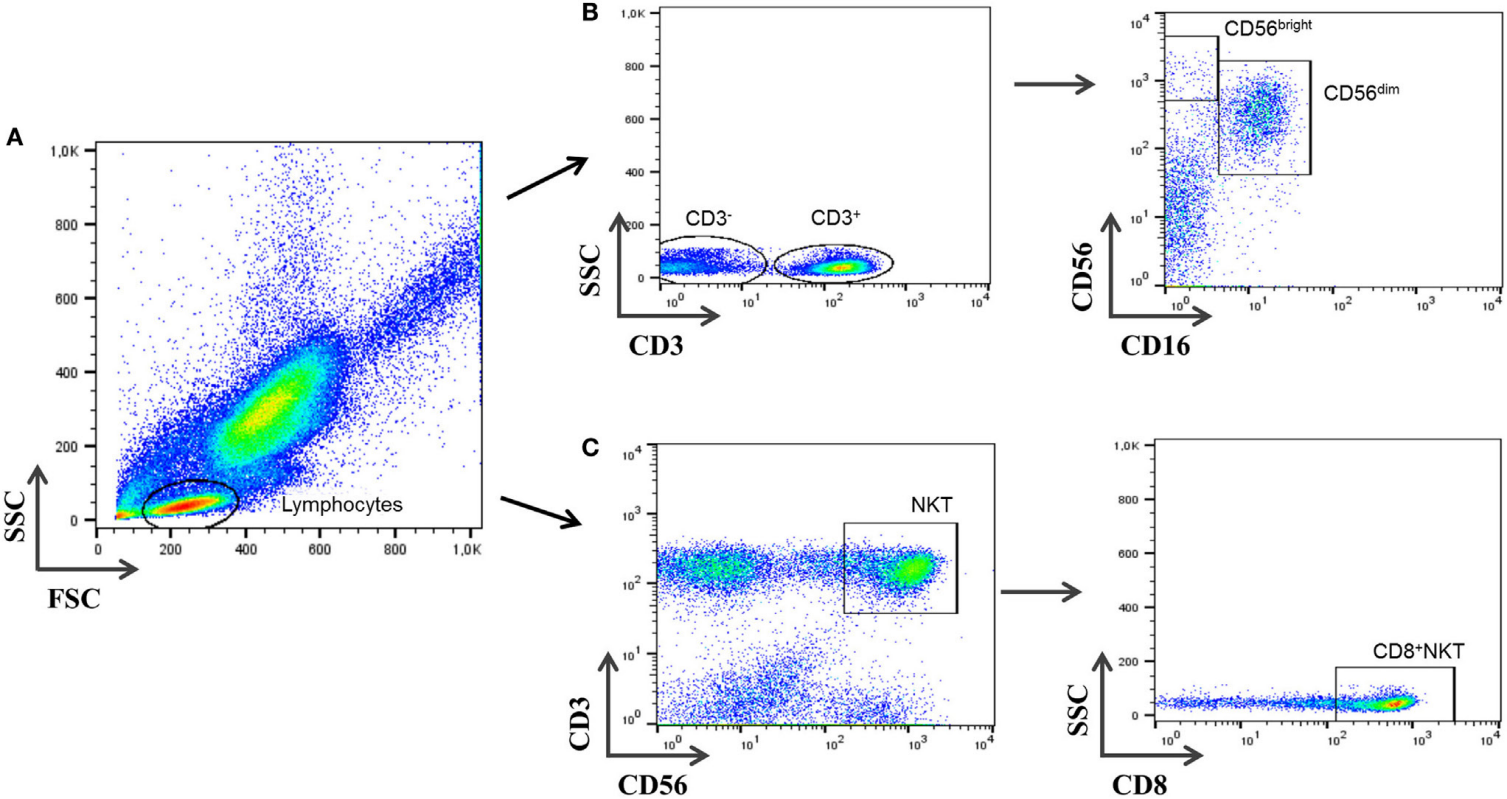
**Gating strategy for natural killer (NK) cells’ subsets and CD3^+^CD56^+^CD8^+^ cells**. **(A)** Peripheral blood events were measured against forward and side scatter parameters, and total lymphocytes were selected. **(B)** Cells negative for CD3 were first selected and further displayed on a plot of CD16 vs. CD56 expression. Cells negative for CD16 and positive for CD56 are CD3^−^CD56^bright^CD16^−^ NK cells and CD3^−^CD56^dim^CD16^+^ NK cells. **(C)** CD3^+^CD56^+^ were analyzed gating on lymphocytes cells and selected from a plot of CD3 vs. CD56. CD3^+^CD56^+^CD8^+^ cells were selected afterward.

Lymphocyte subsets were analyzed using multiparametric flow-cytometry analysis (FacsCANTO, BD Biosciences, San José, CA, USA). Cells were directly stained with the following monoclonal antibodies according to the manufacturer recommendations: CD69-FITC (Mouse Anti-Human CD69, Clone L78; BD Biosciences), CD16-PE (Mouse Anti-Human CD16, Clone B73.1; BD Biosciences), CD3-PerCP (Mouse Anti-Human CD3ϵ, Clone SK7; BD Biosciences), CD56-APC (Mouse Anti-Human CD56, Clone NCAM16.2; BD Biosciences), and CD8-APC-Cy7 (Mouse Anti-Human CD8α, Clone SK1; BD Biosciences). IgG isotypic controls (BD Biosciences) were also tested to determine non-specific staining. Cells were incubated and protected from light at room temperature (RT) for 20 min. Afterward, cells were lysed (FACSTM-Lysing Solution; Becton Dickinson, San Jose, CA, USA), incubated again for 15 min in the dark, and then removed and washed with 2 mL phosphate-buffered saline. In the last step, a 6-color analysis was carried out using FACSCANTO flow cytometer (Becton Dickinson). Cell-Quest research (Becton Dickinson) and FlowJo (Tree Star, Ashland, OR, USA) software were used for the analysis. The gate was set for both FSC and SCC and included lymphocytes (Figure 1). A total of 20,000 events in the lymphocyte gate were acquired for each sample. After further gating on CD3^−^ cells, the percentage of CD3^−^CD56^bright^CD16^−^ and CD3^−^CD56^dim^CD16^+^ NK cell subsets was determined. CD3^+^CD56^+^CD8^+^ cells were analyzed in parallel on total lymphocytes. Although cell populations other than NKT cells might be included in the analysis, as minority subset of γδ-T cells, which represents less than 1% of total CD3^+^CD56^+^CD8^+^ cells (data not shown), non-significant differences were observed in γδ-T cells among all groups studied. Differences in CD3^+^CD56^+^CD8^+^ cells would be ascribed to the CD8^+^NKT-like population. To avoid controversy and enhance accuracy of the nomenclature, we will use the term CD3^+^CD56^+^CD8^+^ herein for this subset instead of “CD8^+^ NKT-like cells.” The CD3^+^CD56^+^CD4^+^ T subpopulation was not considered for the analysis due to its very low percentage of the population studied. All NK and CD3^+^CD56^+^CD8^+^ subsets are given as percentage of total lymphocytes.

### Progesterone and Estrogen Hormonal Quantification

Blood samples were obtained around 8:30 a.m. Serum was separated in a refrigerated centrifuge and stored at −80°C until use. Serum progesterone and estrogen levels were determined in the luteal and follicular phases of the menstrual cycle of both healthy and MS women and men groups by a chemiluminescence assay (Immuno I, Bayer, Germany) following the manufacturer’s instructions.

### Statistical Analysis

Descriptive data are presented as median (interquartile range). When multiple groups with continuous outcomes were compared, the non-parametric Kruskal–Wallis rank sum test was used, followed by pairwise Mann–Whitney tests if the former indicated significant differences. Correlations were assessed using Spearman correlation (rs) coefficients. Data were analyzed with SPSS v.19 (Chicago, IL, USA) and GraphPad Prism software (CA, USA). A *p*-value less than 0.05 was considered as statistically significant.

## Results

### NK and CD3^+^CD56^+^CD8^+^ Cell Subsets’ Changes within the Menstrual Cycle

As previously reported (29), no significant differences neither for the values of total NK cells nor for any of the NK cell subsets between the days 1–3 and 14 of the menstrual cycle of the control group of normal fertile women were found (data not shown). In parallel, no statistically significant differences in the percentage of CD3^+^CD56^+^CD8^+^ cells during the two phases of menstrual cycle were observed. As a conclusion of this part, the study of NK and NKT cells could be performed at any time-point of the menstrual cycle. As expected, sex hormone levels significantly increased on day 14 of the menstrual cycle with respect to day 1–3 (*p* < 0.001): estrogens [39.6 (33.2–54.6) vs. 168.9 (100.0–584.7)] and progesterone [0.4 (0.3–0.6) vs. 3.3 (1.3–7.3)].

### NK and CD3^+^CD56^+^CD8^+^ Cell Subsets’ Changes during Pregnancy

**Figure 2:**
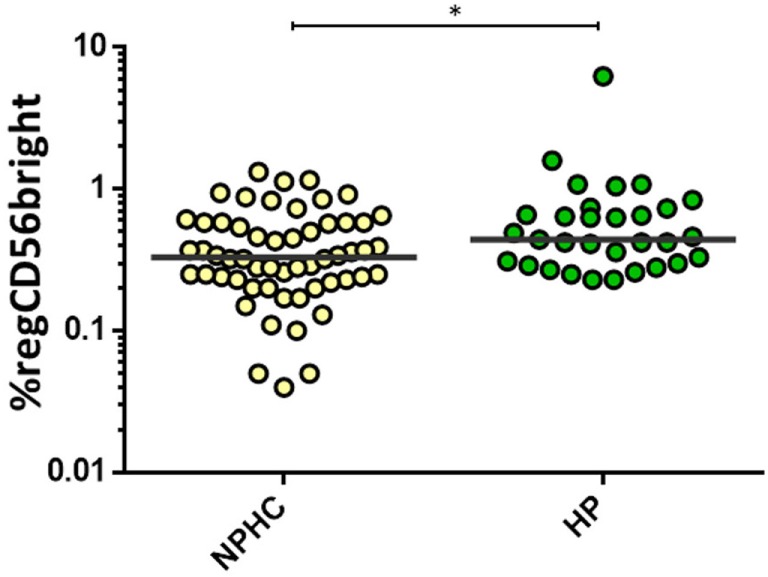
**Percentage of regCD56^bright^ in non-pregnant healthy control women (NPHC) (*n* = 32) and HP (*n* = 13)**. The bar represents the median value, and individual dots indicate single donor values. Mann–Whitney statistical test was used for calculation of the reported *p*-value. *p = 0.015 was considered to be statistically significant.

Healthy pregnant women (HP) showed an increased proportion of the regulatory CD56^bright^ subpopulation [0.44 (0.3–0.73) vs. 0.33 (0.23–0.58); *p* = 0.015] compared to the non-pregnant healthy control women (NPHC) (Figure 2). No differences were observed in CD3^+^CD56^+^CD8^+^ in HP and NPHC.

**Figure 3:**
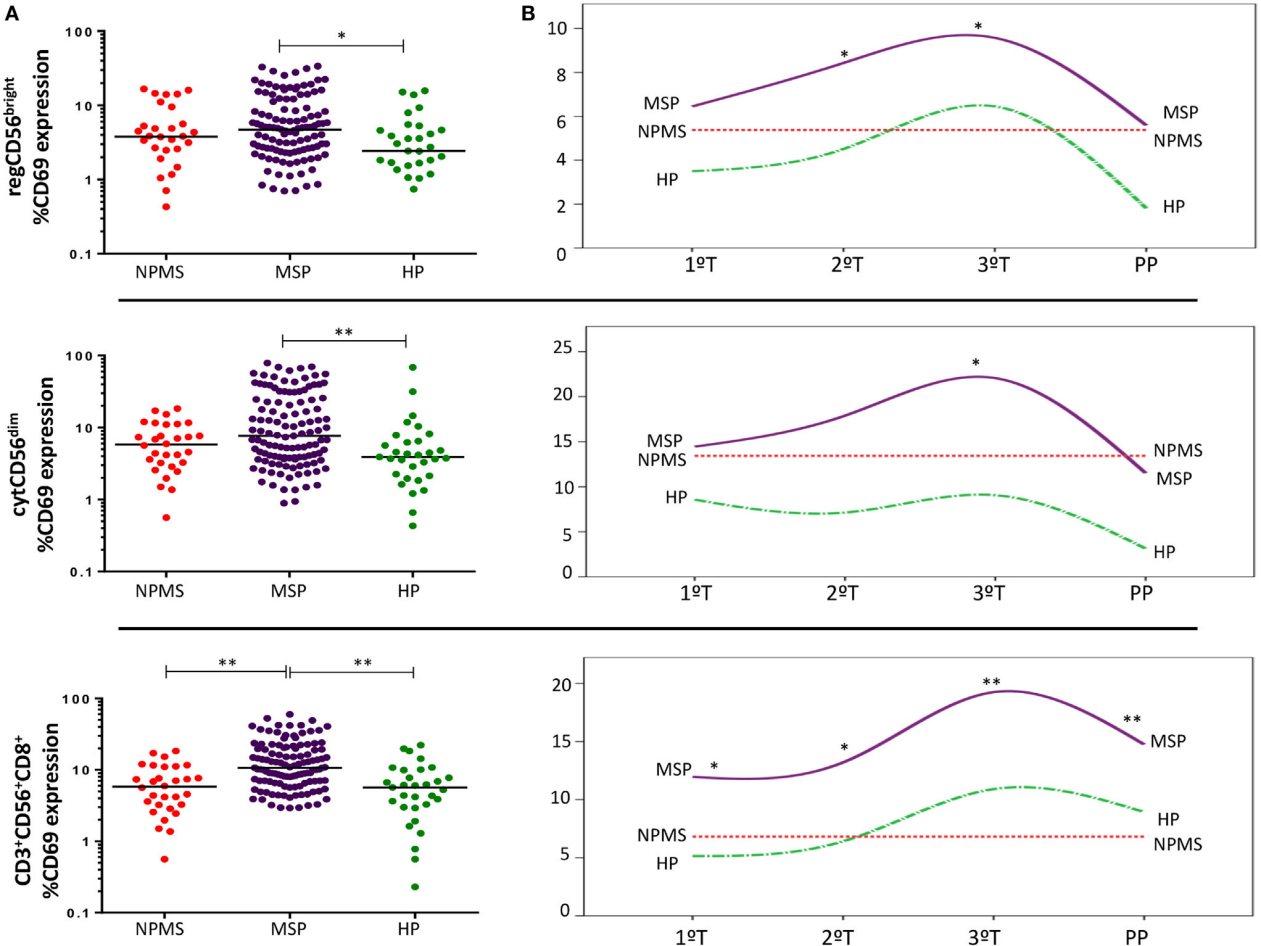
**(A)** Early activation marker CD69 expression by peripheral blood regCD56^bright^ (CD3^−^CD56^+^CD16^−^), cytCD56^dim^ (CD3^−^CD56^+^CD16^+^), and CD3^+^CD56^+^CD8^+^ cell subsets throughout pregnant multiple sclerosis (MS) women (MSP; *n* = 30), non-pregnant MS women (NPMS; *n* = 30), and healthy pregnant women (HP; *n* = 13). The bar represents the median value, and individual dots indicate single donor values. **(B)** CD69 expression longitudinally studied during each trimester of pregnancy (1T, 2T, 3T) and postpartum phase in pregnant women. *Y*-axis represents the media values. Mann–Whitney statistical test was used for calculation of the reported *p*-value. Also, *p* < 0.05 was considered to be statistically significant. Statistical significance is marked as *p < 0.05 and **p < 0.001.

This pattern was not evident in MS pregnancies. Instead, MSP showed a marked early activation of the three NK/NKT populations studied. The most remarkable finding was the significantly increased CD3^+^CD56^+^CD8^+^ activation compared with non-pregnant MS women (NPMS) (*p* < 0.001) and even with HP (*p* < 0.001) (Figure 3A). This increase remained statistically significant and progressed during all the trimesters (T) of gestation and at postpartum (PP) with respect to NPMS (1T: *p* = 0.018; 2T: *p* = 0.004; 3T: *p* < 0.001; PP: *p* = 0.001, respectively). Further, activation of CD3^+^CD56^+^CD8^+^ cells was even higher in MSP than HP in the first two trimesters of gestation (*p* = 0.004; *p* = 0.015, respectively). Specifically, during the second and third trimesters of gestation, there was an increased activation of regCD56^bright^ NK in MS pregnancy compared to NPMS (2T: *p* = 0.063; 3T: *p* = 0.037) and even higher than HP during the second trimester (*p* = 0.033). Only at the third trimester, there was a slightly higher activation of the cytCD56^dim^ NK subpopulation with respect to NPMS and HP (MSP vs. NPMS: *p* = 0.019; MSP vs. HP: *p* = 0.05) (Figure 3B). No significant differences in the proportions of cytNK, regNK, and CD3^+^CD56^+^CD8^+^ cells’ subsets were observed between MSP and NPMS and between MSP and HP.

Regarding sex hormone levels, we did not observe differences during pregnancy between MSP and HP. However, a significant decreased estrogen levels in MSP with respect to HP was shown during the PP (*p* = 0.04). No correlation was showed among NK and NKT cell subsets and sex hormones.

### Gender Effects on NK Cells

To evaluate gender effects, we analyzed global differences between non-pregnant women and men in both MS and healthy groups. Although estrogen levels have a significantly different distribution in women and men (healthy: *p* < 0.001; MS: *p* = 0.002), no gender differences were observed in the proportions of any NK and NKT populations studied in neither healthy nor MS patients.

**Figure 4:**
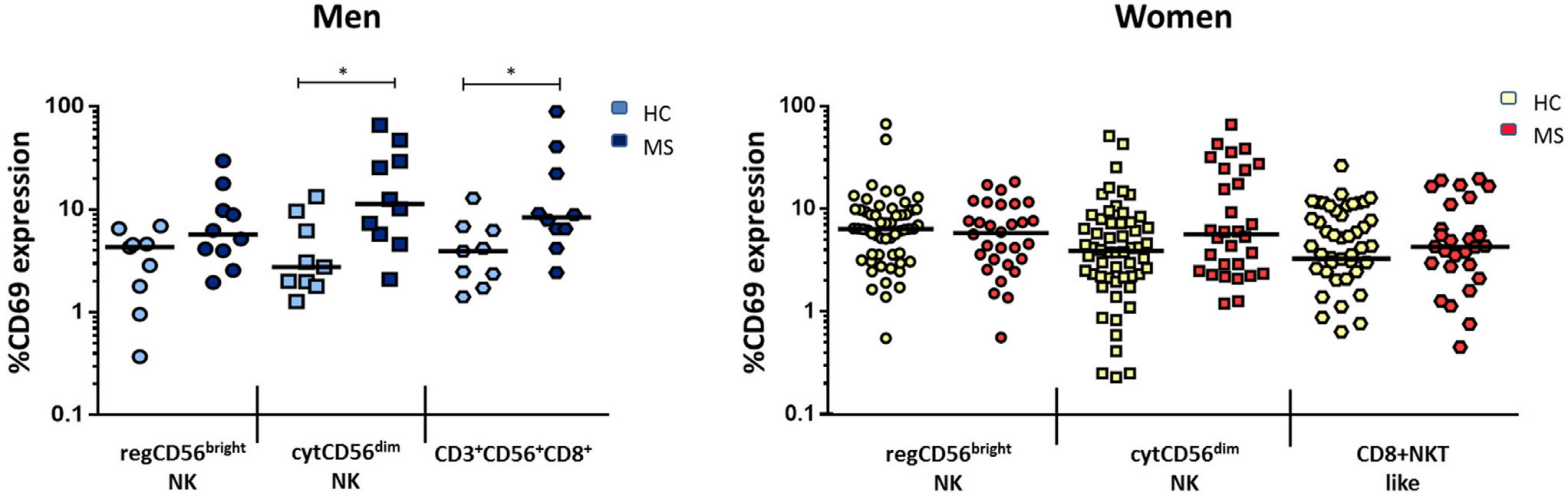
**Expression of CD69 (percentage) of natural killer (NK) subsets regCD56^bright^ (CD3^−^CD56^+^CD16^−^), cytCD56^dim^ (CD3^−^CD56^+^CD16^+^), and CD3^+^CD56^+^CD8^+^ cells in men (left) [HC: *n* = 9, multiple sclerosis (MS): *n* = 10] and women (right) (HC: *n* = 32, MS: *n* = 30) MS compared to healthy individuals**. Mann–Whitney statistical test was used for calculation of the reported *p*-value. The bar represents the median value, and individual dots indicate single donor values. Also, *p < 0.05 was considered to be statistically significant.

There were, however, differences when comparing subsets between MS and the healthy group when considered by gender: when comparing with healthy men, MS men showed a significantly increased activation of both cytCD56^dim^ NK and CD3^+^CD56^+^CD8^+^ cells. The most activated subset was cytCD56^dim^, with a fourfold increase in CD69 expression [11.34 (5.45–33.94) vs. 2.76 (1.89–7.90), *p* = 0.013]. Also, MS men had significantly higher activated CD3^+^CD56^+^CD8^+^ cells than healthy men [8.39 (5.89–27.02) vs. 3.93 (2.04–6.53), *p* = 0.017] (Figure 4), suggesting its relevance in MS pathophysiology.

**Figure 5:**
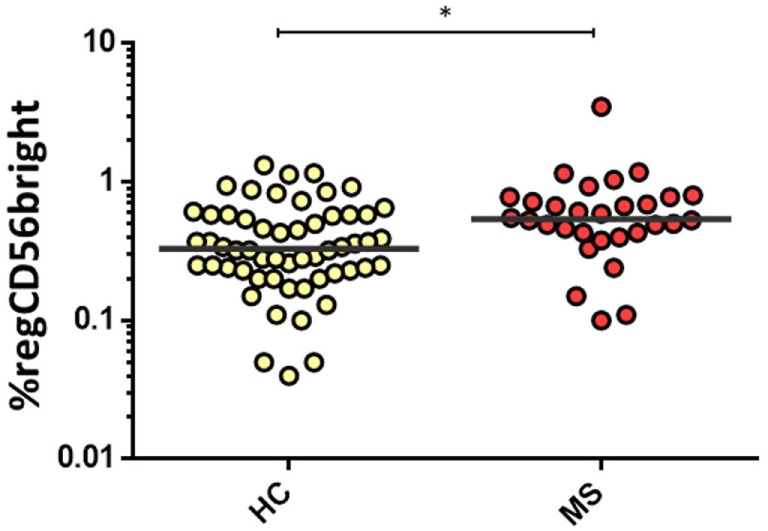
**Percentage of regCD56^bright^ in women from HC (*n* = 32) and multiple sclerosis (MS) (*n* = 30) groups**. The bar represents the median value, and individual dots indicate single donor values. Mann–Whitney statistical test was used for calculation of the reported *p*-value. Also, *p = 0.003 was considered to be statistically significant.

For women, MS women disclosed significantly higher proportions of the regCD56^bright^ subset compared to healthy women [0.54 (0.42–0.78) vs. 0.33 (0.23–0.58), *p* = 0.003] (Figure 5). There were no significant differences in cytNK and CD3^+^CD56^+^CD8^+^ cells’ proportions between both MS and healthy groups.

**Figure 6:**
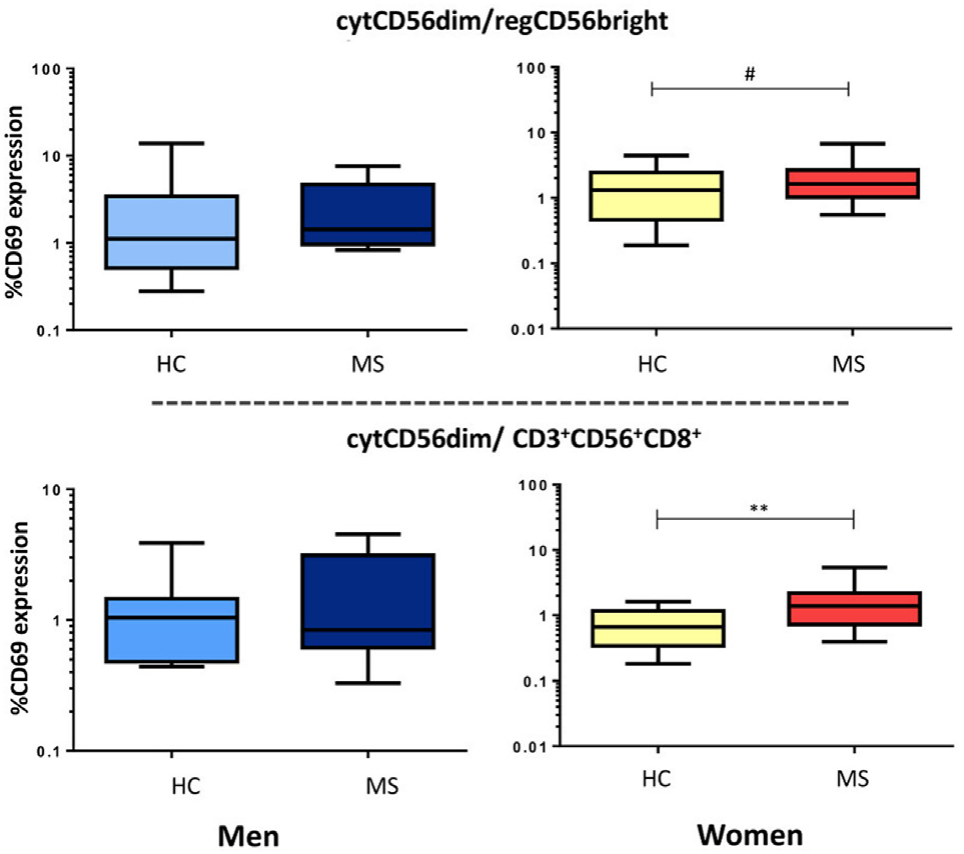
**Ratio of cytCD56^dim^ and regulatory cell subsets (regCD56^bright^ or CD3^+^CD56^+^CD8^+^) activity in multiple sclerosis (MS) men (HC: *n* = 9, MS: *n* = 10) and women (HC: *n* = 32, MS: *n* = 30)**. Data are plotted with box and whiskers (10–90% range), and the bar represents the median value. Also, *p* < 0.05 was considered to be statistically significant. Statistical significance is marked as **p < 0.001 and ^#^*p* = 0.06 (trend).

We also evaluated the balance between cytotoxic vs. regulatory NK cells through the cytCD56^dim^/regCD56^bright^ ratio and the cytCD56^dim^/CD3^+^CD56^+^CD8^+^ cells ratio. We found significantly increased ratios of CD69 expression in MS women with respect to healthy women (cytCD56^dim^/CD3^+^CD56^+^CD8^+^, *p* < 0.001; cytCD56^dim^/regCD56^bright^, *p* = 0.06) (Figure 6).

## Discussion

The most remarkable finding was the significant increase in activation of blood CD3^+^CD56^+^CD8^+^ cells in MS patients during pregnancy with respect to NPMS and even above that of the healthy pregnant women. In addition, healthy full-term pregnancies showed also significantly higher activation of CD3^+^CD56^+^CD8^+^ cells with respect to healthy non-pregnant women. The immunology of pregnancy is characterized by an anti-inflammatory shift paradigm in peripheral blood based on CD4^+^ Treg and Th differentiation toward Th2 and regulatory CD4^+^ T cells (Treg) (30–32). Changes in hormone levels during pregnancy, such as increased estrogens and progesterone, may be responsible for these changes. Thus pregnancy, through the promotion of an immunoregulatory status, has been related to have the most beneficial effects on the severity of several Th1/Th17-driven autoimmune diseases (33).

No reliable studies have shown the role of NKT in the setting of pregnancy and in MS, although our data suggest a potential role of this activated regulatory subset in the pregnancy-related remission of disease activity. Here, we observe that the highest activation status of CD3^+^CD56^+^CD8^+^ cells occurred at the third trimester of gestation, when sex hormones reach their peak, and thereby would coincide with the reported strongest decrease in relapse rate in MS patients (34). Further, persistent although lower activation of CD3^+^CD56^+^CD8^+^ cells was observed at PP, when the probability of MS relapse increases. The pattern of activation of CD3^+^CD56^+^CD8^+^ cells was quite similar to that reported by our group and others for Treg, which have a peak during the second and third trimesters and declining at PP (35), which could reflect their possible implication in MS activity during pregnancy and PP. Regulatory function of CD8^+^NKT cells has been previously reported in several disease mouse models characterized by Th1 immune responses through the production of a Th2-type cytokine profile (24, 36). The changes observed here, especially the high activation status of the regulatory subsets, lead us to speculate that CD8^+^NKT cells could play an important role in modulating T cell responses and in ameliorating MS during pregnancy.

Epidemiological and clinical data clearly underline the sexual dimorphism in MS incidence and disease course. This fact directs to the influence of hormones on immune cells, and estrogens are known to exert opposing and dose-dependent effects on the immune response. There is controversy about specific effects of sex hormones on NK activity (37). In one hand, low estrogen levels facilitate a cell-mediated proinflammatory immune response, whereas their relatively high levels (pregnancy) promote anti-inflammatory Th2 and Treg responses (38). Our findings showed no differences between the follicular and ovulatory phases in the activity or proportions of the three NK and CD3^+^CD56^+^CD8^+^ populations studied in healthy subjects. Concerning gender-based differences, our results showed no differences in peripheral NK or CD3^+^CD56^+^CD8^+^ cell numbers or activity between women and men in healthy and MS groups. Moreover, we did not observe correlation among sex hormones estradiol and progesterone with NK and CD3^+^CD56^+^CD8^+^ cells’ subsets. Reported studies on the fluctuation of NK cells subsets during the menstrual cycle are heterogeneous, showing no changes during the cycle, increase, or even reduction in the luteal phase (37, 39). These results may in part be due to varying definitions of the NK populations and methodologies to define these cells and to measure activity.

However, differences between MS patients and healthy controls differentiated by gender were remarkable. Our findings showed increased activation of the cytotoxic CD56^dim^NK subset in men affected of MS with respect to healthy men. These major increase of the cytotoxic NK subset studied would be compatible with previous results reporting increased circulating cytCD56^dim^ NK levels expressing perforin in primary progressive MS patients, a clinical form with men’ predominance (40), which might suggest a role of this NK cell subset in the pathophysiology and progression of the disease. The increased activation of the regulatory CD3^+^CD56^+^CD8^+^ subset reported in our series of MS men could be reflecting a compensatory activation to control the inflammatory activity. On the other hand, MS women show an increased proportion of regCD56^bright^ NK cells compared to healthy women. Thus, the increased activation of the cytotoxic CD56^dim^ NK subset in men, and the increased proportion of regCD56^bright^ NK cells in women, would be in line with the sex differences reported in the clinical course of MS. Although prevalence in women is higher, there is evidence that women generally have an earlier onset of disease, slightly lower prevalence of PP-MS, and show in general less progression of disability than men (41).

Particular NK cell subtypes may have different roles in controlling CNS inflammation in MS patients, and the balance between immunity and tolerance would be orchestrating the adaptive autoreactive response. MS men showed an increased ratio of cytotoxic NK vs. regulatory NK/NKT cells’ activation; whereas MS women showed a significantly increased proportion regCD56^bright^ NK cells than healthy women. However, the immune balance between cytotoxic and regulatory NK cells was highly increased in MS patients, more marked in women. In terms of NK cells’ functions, we could speculate that more than a defective regulatory response, an imbalance due to enhanced cytotoxic activity could contribute to the MS pathological processes.

Understanding the distribution and function of NK and NKT cells’ subsets related to gender disparity and the effects during pregnancy status in MS may help to have a global vision and to fully understand the implication of innate immunity on MS. Differences observed and beneficial effects of pregnancy in MS highlight the relevance of NKT cells. Further studies on CD8^+^ NKT cells function and their role in pregnancy beneficial effects on MS are warranted to move forward more effective MS treatments. NKT-like cells would be potential therapeutical targets in MS as well as therapies that enhance their numbers or activation.

## Ethics Statement

All subjects gave written informed consent in accordance with the Declaration of Helsinki. The protocol was approved by the Ethical and Scientific Committees of the Hospital.

## Author Contributions

CA recruited and followed patients, wrote the draft the manuscript, and performed critical revision; LF-P analyzed data, wrote the draft the manuscript and figures; BA acquired data; MT-A and RR-M acquired data, analyzed and revised the manuscript; SS-R designed concept of the research and experiments, analyzed data and critical revision, and wrote the manuscript.

## Conflict of Interest Statement

The authors declare that there is no conflict of interest regarding the publication if this paper. The funders had no role in study design, data collection and analysis, decision to publish, or preparation of the manuscript.
